# Centennial Review: Trace mineral research with an emphasis on manganeseDedicated to Dr. Roland M. Leach, Jr.

**DOI:** 10.1016/j.psj.2021.101222

**Published:** 2021-04-27

**Authors:** Michael S. Lilburn

**Affiliations:** Department of Animal Science, The Ohio State University, Wooster, OH 44691, USA

**Keywords:** embryo, manganese, perosis, proteoglycan, skeleton

## Abstract

A century of publications in the *Poultry Science* journal is celebrated with Centennial papers. It is relevant, therefore, to explore trace mineral (**TM**) research with an emphasis on manganese and selected aspects of skeletal development. Some of the initial observations on the topic appeared in the earliest volumes of our journal. Published studies in the late 1920’s and 1930’s confirmed the importance of the diet and *unidentified* organic (i.e., vitamins) and inorganic nutrients (i.e., TM) relative to skeletal development. The early nutrition research emphasized requirement studies, the search for unknown factors to alleviate recognized deficiencies, and lastly important nutrient interactions, especially in the gut. This review will discuss TM research with an emphasis on manganese (**Mn**). Some of the fundamental discoveries on the mechanisms underlying embryonic and post-hatch skeletal development led directly to research directed at the role of Mn in the synthesis of the epiphyseal matrix. The TM research agenda today is considerably different with respect to all trace nutrients and is largely driven by gut health, antibiotic free production, food safety and environmental outcomes. A significant proportion of the published research over the last 2 decades has focused on the form (i.e., organic, inorganic) of a given TM relative to a given physiologic or production response under the pretext that modern commercial genotypes and production realities have changed considerably since the last NRC publication (NRC, 1994). If one closely reviews the more recent scientific literature, however, it could be argued that the term “trace mineral requirement” is often a misnomer. Many of the TM levels recommended or in use today are not the result of quantifiable requirement studies but are often based on efficacy comparisons with the different organic and inorganic forms of commercially available TM.

## INTRODUCTION

An extensive treatise on post-hatch skeletal growth was published by Latimer (1927). During the following decades, there was considerable nutrition research on skeletal development in poultry with the goal of alleviating the prevalent skeletal anomalies of that particular era. (i.e., perosis; rickets). At the time these early reports were being published, essential vitamins and trace minerals (**TMs**) were still largely unknown. The design of a given experiment to study a specific clinical problem often had treatments which were simply different levels or combinations of ingredients with curative properties (i.e., rice bran, wheat germ). The early delineation of what we now know as essential vitamins (organic) and TMs (inorganic) were often defined by their presence or absence in the ash of curative ingredients. As will be noted later in this paper, Gallup and Norris (1939a) observed that while 50 ppm Mn would decrease the incidence of perosis in New Hampshire chicks, only 30 ppm was needed by leghorn chicks. This was one of the first instances in which genotype differences were included in the discussion on nutritional requirements. Creek et al. (1960) would later show that body weight was a determinant of the severity of perosis. In a paper considered to be one of the seminal publications on skeletal growth in poultry, Church and Johnson (1964) showed that within a genotype, body weight and bone growth curves are very similar through 5 days post-hatch after which the inherent variability within a population increases. They noted that in both Barred Rock and Leghorn chicks, long bone development was greatest from 0 to 3 weeks and overall skeletal maturation was accelerated in the heavier, Barred Rock chicks. The critical importance of the first 3 weeks post-hatch, especially in commercial broiler genotypes, was subsequently confirmed by Williams et al. (2000) and Applegate and Lilburn (2002).

It has been well documented that genetic selection has been largely responsible for the improved growth and carcass traits in commercial broilers (Reddy, 1996; Havenstein et al., 2003a,b). Yair et al. (2017) more recently reported that post-hatch differences in genetic growth potential may also be reflected in embryonic skeletal traits, using reciprocal matings between fast and slower growing strains to experimentally equalize egg size. Commercial selection programs in broiler and turkey genotypes have not only changed their growth and carcass characteristics but also the relationships between the latter 2 traits and the physiology of growing lone bones (Leach and Nesheim, 1965; Leblanc et al., 1986; Kestin et al., 1992; Leterrier and Nys, 1992; Pitsillides et al., 1999, Williams et al., 2004; Williams et al., 2004). Changes in carcass conformation and the potential for carryover effects on ambulation in commercial broilers have also contributed to an increased incidence of bacterial chondronecrosis in the tibia and femur (Wideman & Prisby, 2013, Wideman et al., 2014, Wideman, 2016). The resulting lameness from these bacterial challenges have both economic and animal welfare consequences. In addition to the aforementioned genotype changes, nutrition and management approaches have also changed considerably over the years, largely the result of the paradigm shift to antibiotic free production and the advent of third party welfare audits of production facilities.

The citations often referenced in the published literature and/or recent reviews on TM supplementation will often include publications from the 1960’s and 1970’s. A critical review of the literature, however, shows that papers published 20-30 years earlier paved the way for the subsequent discoveries on the essentiality of key TM. The perceived lack of appreciation for the foundation papers underlying our present understanding of TM was the motivation for the writing of this review. It seemed appropriate to include this as a Centennial paper for Poultry Science given that the initial research discussed herein was conducted and subsequently published in some of the earliest issues of the journal.

### Development of Defined Diets

The initial, underlying research which established the dietary requirements for many trace nutrients occurred during a time when unidentified factors were starting to be recognized as being important dietary cofactors. It is important to note that during this earlier research period, there was a considerable focus on the development of synthetic or defined diets which largely used chicks as the experimental animal model (Drummond, 1916; Osborne and Mendel, 1918). These defined diets became the research tool used in establishing the importance of many essential micronutrients (i.e., Vitamin A; Hart et al., 1920; 1924) and in the determination of which micronutrients/ingredients were effective in alleviating major nutritional deficiencies (i.e., cod liver oil and rickets; Dunn, 1924). Numerous reports from this era used BW as a primary metric for studying nutrient and ingredient interactions and many of these studies included notations on skeletal issues, often referred to as *“leg weakness”* (Hart et al., 1920, 1922; Johnson, 1925). There were also those research groups that questioned the applicability of synthetic diets in studies relative to optimizing the growth and development of chicks reared in artificial or confined environments (Hogan et al., 1925, 1928; Hogan and Shrewsbury, 1930). It was during this time that McCarrison (1927) reported some of the first observations on a dietary requirement for manganese (Mn) in rats.

### Ingredient and Macromineral Interactions

Norris et al. (1930; 1931) described a “*nutritional paralysis”* of the lower skeleton and noted that dietary supplementation with meat scrap, menhaden fish meal, and dried whale meat did not alleviate this condition and speculated that possibly a third, unidentified essential vitamin in milk was involved. At the time, vitamin B (polyneuritis) and vitamin G (pellagra) were recognized trace factors found in milk and milk products. Hart et al. (1930b) dismissed the idea of an unknown vitamin and countered that the “*nutritional paralysis*” described by Norris had been observed several years earlier in chicks which had consumed “ample quantities of milk”. Chicks with *“range paralysis”* were sent from the Ontario Agricultural College (Professor Graham) to the University of Toronto for bone structure analysis (histology), Ca and P determination (bone, serum) and bone phosphatase levels (Hall and King, 1931). The authors reported that histology, serum and bone measurements were similar with the only abnormal findings being a “subluxation, bowing and rotation” of the bones. The symptoms described in the Norris and Hart reports were similar to the “*range paralysis*” described by Hall and King (1931) and later Wilcke et al. (1933). As the incidence of these long bone anomalies became more common, Titus (1932) suggested that there appeared to be a consistent pattern of adverse skeletal events which he lumped together, coining the term “perosis.” The individual sequential events included an initial enlargement of the hock with hemorrhages in surrounding tissues, curvature of the tibia, and finally a slipping of the gastrocnemius tendon from the underlying articular cartilage, hence the term “*slipped tendon.*” He acknowledged that this sequence of individual pathologies was similar to what had been previously described by Payne (1930). Milby (1934) later reported that the “slipped tendon” was a major causal factor in the structural bending of the bone (i.e., bowed legs) and hypothesized that the degree of tibial curvature was related to the severity of the deformity.

In an interesting series of experiments reported by McFarlane et al. (1931), individual animal protein sources (meat meal, buttermilk powder, fish meal, gelatin, cod liver meal, casein plus yeast derivatives) were incorporated into otherwise defined diets to compare their efficacy in supporting chick growth. All the diets were standardized to 18% CP and none of the “animal protein” sources alone would support acceptable chick growth without accompanying “leg weakness.” Visible deficiency symptoms associated with individual protein sources varied with the age of the chicks. The basal diet with fish meal was similar to that of Plimmer et al. (1927) who had successfully reared chicks with no reported skeletal issues. One noted difference between the 2 reports, however, was the use of dextrin as a carbohydrate source by McFarlane et al. (1931) whereas Plimmer et al. (1927) had used white rice. McFarlane et al. (1931) subsequently included white rice (~50%) in a basal diet replacing dextrin and this improved growth and markedly decreased leg issues and mortality. These authors limited their conclusion to some component of “rice protein” in eliciting or facilitating the beneficial response. The results of McFarlane et al. (1931) supported previous observations by Hogan et al. (1925) who reported considerable improvements in chick growth through maturity when corn starch was replaced by polished rice in semi-purified diets.

The published results of McFarlane et al. (1931) increased research interest relative to identifying which ingredients or ingredient fractions could potentially reduce the incidence of “*leg weakness*”. Bethke et al. (1931) reported that wheat middlings with germ and wheat germ alone improved growth but did not cure perosis whereas dried pork and beef liver were effective. The annual meeting of the Poultry Science Association (PSA) became an increasingly important forum for the sharing of data and these presentations became the basis for much of the early published literature. At the 23^rd^ annual PSA meeting, 3 papers were presented on the “*slipped tendon and hock disease.*” Hunter et al. (1931) reported that oats and oat fractions alleviated the condition in leghorn pullets while Titus and Ginn (1931) observed a similar effect with dietary inclusion of 10% rice bran. Buckner et al. (1931) discussed and contrasted the slipped tendon condition with deformed metatarsal bones resulting from supplementation with different combinations of magnesium carbonate and tricalcium phosphate. Titus (1932) reported on a “chance observation” that showed the effectiveness of rice bran in reducing the incidence of perosis with subsequent experiments suggesting that 6% rice bran was the minimum effective supplementation level. It is possible that a small amount of rice bran in with the white rice may have contributed to the beneficial effects previously attributed to McFarlane et al. (1931). Sherwood and Couch (1933) repeated the Titus (1932) experiments and reported that replacing corn with 5 to 10% rice bran or 20% wheat gray shorts would reduce the percentage of slipped tendons. At the 25th annual PSA meeting, Branion (1933) reported that chicks fed a nutritionally adequate diet containing corn as the only cereal resulted in considerable bowing of the long bones with a high proportion of slipped tendons, similar to what was observed by Hogan, et al. (1925). The incidence of the condition decreased with dietary inclusion of wheat germ or oat hulls/germ and the positive effects of wheat germ were later confirmed by Graham et al. (1934), contrary to what had previously been reported by Bethke et al. (1931). This latter point emphasizes some of the contradictions in the early literature that may have been simply the result of the variability due to sourcing of the ingredients used in the early research. It is also interesting to note that in studies conducted almost 60 years later (Halpin and Baker, 1987), the not as yet defined neutral detergent fiber fraction in common ingredients was reported to be a significant modulator of Mn absorption.

### The Move Beyond Bone Mineralization

The recognition that previously unknown dietary factors could be influencing skeletal development was based largely on secondary observations in studies which emphasized the roles of Ca, P, and the Ca:P ratio in bone mineralization (Hart et al., 1930a). There was a growing consensus that the total mineral content (Ca, P) and source of minerals (i.e., bone meal) in the diet were positively related to the incidence of perosis with no effects on bone morphometry or ash content (Hall and King, 1931; Herner and Robinson, 1932; Holmes et al., 1933). Payne et al. (1932) reported that a mix of minerals including calcium phosphate and calcium carbonate resulted in the same incidence of perosis as steamed bonemeal. Parkhurst and McMurray (1933) observed that perosis was negligible if meat and bone meal levels were low or calcium carbonate was the primary source of calcium. Milby (1933) ran a statistical analysis with single and multiple correlations using published data available at the time (Mussehl et al., 1927; Hunter and Funk, 1931; Payne et al., 1932; Titus, 1932). The correlation (*r* = 0.630) between percentage slipped tendons and total dietary P was significant and as good a predictor as the combination of all the factors tested (dietary Ca, protein, P, bone ash). Insko et al. (1934) observed that simply widening the Ca:P ratio did not increase the incidence of slipped tendons but when bone meal was used to increase P levels as part of the ratio adjustment, there was an increased incidence, supporting the correlation analysis by Milby (1933). At the 27th PSA meeting (1935), Hammond (1935) noted that dietary inorganic P vs. organic or total P had the highest correlation with the incidence of perosis and there was a negative correlation between perosis and Ca. It is interesting to note that the form of P in the diet (inorganic, organic) reported by Hammond (1935) was several years before some of the initial reports in the literature on the effects of phytate on P digestibility in poultry fed vegetable based diets (Common, 1939; Lowe et al., 1939). Wilgus et al. (1935) discussed how monosodium phosphate and steamed bone meal were causative factors for perosis while among the practical ingredients, wheat germ was effective in reducing the incidence, again in support of earlier published data (Branion, 1933; Graham et al., 1934). Schaible et al. (1935) supported the growing consensus that bonemeal and calcium phosphate predisposed chicks to perosis whereas 5% added oyster shell (calcium carbonate) decreased the incidence. This paper was also one of the first to discuss the concept of acid/base balance and the source of protein as being predisposing factors, particularly in diets high in total mineral content. These authors also observed that some chicks were hatched with a malady similar to perosis or acquired it early in life if the diet was low in total minerals.

### The Importance of Manganese

In their paper initially describing “*nutritional paralysis,*” Norris et al. (1930) commented that one goal of their research was to establish a defined reference diet that could subsequently be used for Ca and P requirement studies. At the seventh World's Poultry Congress, Wilgus et al. (1939) discussed the happenstance observation that an impurity present in a particular source of monocalcium phosphate prevented rather than exacerbated the incidence of perosis (Wilgus et al., 1937a). The impurity was subsequently shown to be manganese (Mn) and shortly thereafter it was reported to be a major dietary factor predisposing chicks to the onset of perosis if fed Mn deficient diets (Wilgus et al., 1936; Wilgus et al., 1937b). It was suggested that 35 ppm Mn was needed to minimize perosis and this was one of the first actual requirements published for a TM (Wilgus et al., 1936; 1937a). Wilgus et al. (1937a) later reported that supplementing Zn and Al would also moderately reduce the incidence of perosis but a combination of Mn, Fe, and Al was the most effective dietary treatment, particularly in diets with minimal levels of Ca and P. These authors subsequently suggested that the preventive effects of different cereal grains were a function of their Mn content. At the 1936 PSA meeting, Heller and Penquite (1936) presented several years of data on diet/skeletal weakness and confirmed the link between high mineral levels and perosis. They noted that after encouragement from Wilgus to include supplemental Mn in subsequent studies, they too were able to confirm the beneficial effect of Mn (Heller and Penquite, 1937). These authors also observed that 15% rice bran was not effective in reducing perosis in one particular study but in a follow-up experiment, the dietary inclusion of the ash from 70 lb of rice bran was as effective as 0.02% MnCO_3_ or 0.02% MnCl_2_ in reducing perosis, thus supporting the earlier data from Titus (1932) and Sherwood and Couch (1933). Heller and Penquite (1937) concluded there appeared to be a positive relationship between the Mn content of ingredients and the overall incidence of leg abnormalities including perosis. These authors also warned, however, of potential toxicity issues when high levels of Mn (1.0 % MnCO_3_) were included in the diet. Clifcorn et al. (1938) developed a defined diet devoid of grains and grain byproducts and this diet was subsequently used by Wiese et al. (1938) to determine which rice bran fractions and levels of supplemental Mn might be effective in reducing the incidence of perosis. Insko et al. (1938) fed diets with low or high Ca and P levels in combination with 0 or 30 ppm supplemental Mn (MnSO_4_). At both 4 and 8 weeks of age, the diet with 30 ppm Mn was the only effective treatment in reducing both average percentage bowed legs and slipped tendons. In a subsequent experiment, the combination of 30 ppm Zn, Al, and Fe was tested alone or in combination with Mn and only diets with supplemental Mn proved to be effective, contrary to the moderately positive effects of Zn and Al reported by Wilgus et al. (1937b). Gallup and Norris (1938) subsequently observed that the slipped tendon and condyle issues observed with Mn deficiency were accompanied by a measurable increase in tibia thickness and decreased tibia length. Caskey et al. (1939) later reported that wing bones in addition to the long bones were similarly affected by Mn deficiency and hypothesized that Ca and P metabolism were dependent upon adequate dietary Mn.

### Manganese Interactions in the Intestine

The late 1930’s was a focused period with respect to research interest in mineral interactions within the intestine relative to their digestibility and subsequent bioavailability. Schaible et al. (1938) published an extensive review of the existing literature on Mn and perosis which included analytical values for the Mn content of multiple ingredients/forages. This review also included 2 interesting references on the location of Mn within different plant species and its importance to plant and potentially animal metabolism (McHargue, 1914; 1922). The location of Mn within the plant could help explain the subsequent observations that the bran fractions from rice and wheat were beneficial at reducing the incidence of perosis. Schaible et al. (1938) developed high mineral (32% corn; 6% bonemeal; 37 ppm Mn) and low mineral (67% corn; 11 ppm Mn) diets which induced perosis and hypothesized these diets either decreased Mn solubility/availability in the gut (bonemeal) or resulted in a frank Mn deficiency (high corn diet). When 30 ppm Mn from different salts was added to the low mineral, high corn diet (11 ppm Mn), 41 ppm total Mn minimized perosis, which was close to the 35 ppm suggested as being adequate (Wilgus et al., 1937b). They also suggested that nutrient requirements derived from experiments with either a high proportion of corn or increased mineral levels be reconsidered if these diets were atypical when compared with practical type diets. Wilgus and Patton (1939) would later support the hypothesis that insoluble Ca phosphates (i.e., steamed bone meal) could precipitate Mn from solution and elicit a deficiency in diets marginal in Mn. The partitioning and subsequent availability of Mn from the liquid and solid phases of the intestinal digesta as affected by Ca and P was subsequently described in great detail by Bandemer and Schaible (1942) and Schaible and Bandemer (1942). These authors also observed that the concentration of Mn in bones from deficient chicks was lower than in normal chicks which supported the conclusions by Gallup and Norris (1938) who had hypothesized that Mn was required for normal bone development.

Within their extensive technical bulletin, Schaible et al. (1938) wanted to test the hypothesis that Mn deficiency induced via macromineral interactions in the gut or Mn deficient grains was the primary causal effect of perosis. These authors injected previously calibrated solutions of Mn (0.8% MnSO_4_.H_2_O) on alternate days beginning at a week of age and this completely eliminated perosis. There were 2 similar papers published at approximately the same time that corroborated these observations (Lyons et al., 1938; Caskey and Norris, 1939). Lyons et al. (1938) showed clearly that Mn injection but not Zn, Al, or Fe was effective at improving BW with almost a complete absence of perosis and reduced “*leg bowing.*” In the study by Caskey and Norris (1939), 15 ppm dietary Mn reduced perosis in chicks fed a control diet (1.0% Ca, 0.5% P) whereas 140 ppm Mn was still not completely effective when chicks were fed diets with increased mineral levels (3.0% Ca, 1.5% P). In a subsequent study, intraperitoneal injection of Mn (10 mg) completely eliminated perosis whereas 20 mg significantly reduced growth. They also cited published rat data in which the negative effect of high soluble Mn intake was negated if the diet was sufficiently high in P (Becker and McCollum, 1938) and also noted that 1,100 ppm dietary Mn did not have any noticeable effects on growth (Gallup and Norris, 1939a). Wilgus and Patton (1939) hypothesized that insoluble Ca phosphate in ingredients such as steamed bone meal might be precipitating Mn in the gut and thereby eliciting a deficiency when Mn levels were marginal, similar to the conclusions by Schaible et al. (1938). Similar macromineral – Mn relationships would be reinvestigated and confirmed by Wedekind and Baker (1990a) approximately 50 years later.

### Perosis and Predisposing Factors Other than Manganese

Over the course of the 1930’s, even as numerous studies demonstrated the benefits of Mn supplementation, it was still not 100% effective in experiments with Mn deficient diets and chicks that were predisposed to the onset of the anomaly. Hogan and Shrewsbury (1930) reported that symptoms of perosis could be alleviated by dietary inclusion of wheat or wheat midds but the ash from these ingredients was ineffective, suggesting that the curative factor(s) was organic. Branion (1933) later observed that wheat germ was also effective in reducing perosis and this group subsequently reported the benefits of an organic component in concentrated extracts of wheat germ distinct from Mn (Van der Hoorn et al., 1938). Wiese et al. (1938) confirmed previous reports that rice bran and supplemental Mn were both effective at minimizing perosis but autoclaved rice bran was much less effective. They suggested rice bran contained a “labile organic factor” which was effective alone or facilitated Mn function. Hogan et al. (1940) confirmed that Mn alone was not sufficient but was completely effective in combination with an alcohol extract of dried liver. From a species comparison standpoint, Jukes (1939) reported that a basal diet containing 0.1% MnSO_4_ eliminated perosis in leghorn chicks but higher dietary levels (0.4% MnSO_4_) or Mn injections were not effective in turkey poults. The observation in poults was supported by Ringrose et al. (1939) and Jukes (1940a,b) subsequently reported that a basal diet with supplemental Mn and 0.1% choline was essential for growth but the same diet with 0.2% choline was needed to reduce perosis. A diet with 25% soybean meal was also noted as having anti-perosis properties in poults (Funk and Kempster, 1940) and this was attributed to the levels of lecithin and choline at this level of soybean meal inclusion. The effectiveness of choline in turkeys was confirmed by Evans et al. (1942, 1943). Jukes (1941a,b,c) subsequently went on to report some of the first experiments in poultry on the potential dietary interrelationships between choline, betaine, and methionine.

Cravens et al. (1944) observed embryonic anomalies associated with biotin deficiency that they referred to as “chondrodystrophy.” The deficient embryos, however, were reported to have clinical symptoms more similar to those observed by Landauer and Dunn (1926) than what was described with Mn deficiency by Lyons and Insko (1937a).

From the perspective of genetics, Serfontein and Payne (1934) suggested there was a genetic predisposition for perosis with “straight legs” and “crooked legs” used as the selection criteria as they best represented the observed clinical symptoms. Gallup and Norris (1939a) noted that in New Hampshire chicks, 50 ppm Mn could significantly reduce but not eliminate perosis whereas a lower level (30 ppm Mn) was effective in leghorn chicks. One seemingly obvious contributing factor to the genotype responses were the differences in growth and skeletal development in leghorn vs. heavier broiler type chicks and years later, Creek et al. (1960) would report that body weight was an important criterion relative to the severity of perosis.

### Manganese, Laying Hens, and Embryonic Skeletal Growth

One of the first written accounts on the avian skeleton was a description of different stages of embryonic bone growth (Johnson, 1883). Fell (1925) published a characterization of cellular changes during embryonic bone development followed many years later by a detailed description of the ontogeny of embryonic bone formation and associated morphology (Pechak et al., 1986a,b). Byerly et al. (1933) observed that embryos from hens fed diets with different non-animal proteins included a number of “short boned individuals.” They subsequently reported that these embryonic skeletal anomalies could be alleviated with the supplementation of either wheat germ/whey or wheat germ/liver to the hen diet (Byerly et al., 1935). The observation of shortened and thickened embryonic long bones was confirmed by Gallup and Norris (1937) and Lyons and Insko (1937a,b). In the latter reports, hens fed a Mn deficient diet (5.5 ppm) had a significantly reduced hatch percentage (< 10%) and a number of embryos with shortened and thickened long bones. This condition was referred to as “chondrodystrophy,” so named after the mammalian condition known as *Chondrodystrophia foetais* (Landauer and Dunn, 1926; Hutt and Greenwood, 1929). If eggs from hens fed a deficient diet were injected with Mn prior to incubation, embryonic development was normal and injections of Zn or Fe were without effect. Following the reports by Lyons and Insko (1937a,b), the terms chondrodystrophy and perosis began to be used interchangeably even though they were embryonic and post-hatch skeletal anomalies, respectively. Schaible et al. (1938) subsequently confirmed that there were no differences between controls and diets with >39 ppm Mn.

Gallup and Norris (1939b) suggested that 53 ppm Mn was adequate for normal fertility and hatchability and dry yolk Mn (mg/100 g) increased linearly when dietary Mn was increased to 500 and 1,000 ppm, respectively. These authors also noted that while hatchability was significantly decreased in hens fed Mn deficient diets, the chicks that did hatch were not predisposed to develop perosis if subsequently fed a Mn adequate diet. Caskey et al. (1939) confirmed that Mn supplementation to both hens and chicks was needed for normal long bone (tibia, femur, metatarsus) and wing bone (humerus, radius, ulna) growth. These authors were also careful to differentiate between the specific need for Mn in skeletal development vs. the broader Mn requirement for body weight. Caskey and Norris (1940) subsequently showed that these embryonic skeletal effects in afflicted chicks were not reversed with a Mn adequate diet post-hatch. These studies were soon supported by studies linking Mn deficiency with skeletal issues in swine (Miller et al., 1940) and albino rats (Barnes et al., 1941). The observations on reproduction and embryonic development in chicks came several years after Mn was deemed essential for normal reproduction in both male and female rats (Kemmerer et al., 1931; Orent and McCollum; 1931; Daniels and Everson, 1935).

The studies by Byerly et al. (1935), Lyons and Insko (1937a) and preliminary observations by Norris and Caskey (1939) included descriptions of chicks from Mn deficient hens exhibiting head retraction or “ataxia.” Caskey et al. (1944) reported that the incidence of ataxia ranged from 2.3 to 11.7% (3 separate hatches) and there was no evidence of notable differences in brain histology. This condition was similar to the loss of coordination observed by Shil and McCollm (1943) in young, Mn deficient rats and it was subsequently linked to impaired otolith development in the inner ear (Hurley and Everson, 1959; Asling et al., 1960; Everson et al., 1959; Shrader and Everson, 1967). Erway et al. (1970) would later show that mice with ataxia born from Mn deficient dams had partial or complete absence of the otolith bodies of the inner ear and they noted this was also true for ataxic chicks.

The recognized essentiality of Mn for normal embryonic development ushered in a research era which emphasized the understanding of those mechanisms underlying the dietary requirements for Mn and other TM. There have been several reviews over the last 25^+^ years that have discussed different aspects of TM nutrition and storage (Richards and Steele, 1987; Richards, 1997; Miles, 2001; Torres and Korver, 2018). With respect to Zn and Mn, Bellairs et al. (1972) showed that a granule subfraction in the yolk was the primary storage site in the egg and this supported previous observations for Mn by Gallup and Norris (1939b) and later confirmed for Mn and Zn by Grau et al. (1979). Phosvitin, derived from vitellogenin, is a major component of yolk granules and as a phosphoprotein, contains a high proportion of phosphate groups. It is not surprising, therefore, that yolk granules would be rich in TM. Review papers by Naber (1979) and Angel (2007) are often cited in the current literature to support the hypothesis that the hen diet is not an effective way to increase egg/yolk TM levels. Naber (1979), however, included Mn in a list of nutrients that could be positively affected by diet while Zn was on the list referred to as having no data available. Stahl et al. (1988) subsequently showed that Zn supplemented as the inorganic sulfate would significantly increase egg concentrations and even very high levels (1861 ppm) did not affect egg size or production.

In commercial practice today, nutritionists can choose between “inorganic” TM (i.e. sulfates) or “organic” chelates. Mabe et al. (2003) fed hens diets with 0, 30, and 60 ppm supplemental Zn and Mn as either an inorganic sulfate or amino acid chelate. There was a stepwise increase in yolk Mn with increasing level of supplementation but no difference between the 2 Mn sources. The amino acid-Zn chelate increased yolk levels with 30 ppm supplementation and while 60 ppm resulted in an additional incremental increase from both sources, there was no difference between them. With respect to Cu, Elvejem et al. (1929) did not see any changes in yolk Cu levels with dietary supplementation. Many years later, Guclu et al. (2008) also observed no significant differences in yolk Cu from hens fed 150, 300, or 450 ppm dietary Cu which, it should be noted are levels well above the NRC (1994) recommendation of 8 ppm.

Dewar et al. (1974) was among the first to describe the pattern of TM transfer from the egg to the embryo. In that study, hens were fed a basal diet containing Zn (72 ppm), Mn (81 ppm), and Cu (22 ppm). Individual mineral concentrations (*ug/g dry embryo)* decreased precipitously from ED5 to ED10 of incubation followed by a continual but less dramatic decrease between ED10 and ED18 d. The authors calculated the transfer efficiency from the egg to the embryo at 69.8% (Cu), 50.7% (Zn) and 36.2% (Mn) and noted that the latter value for Mn was considerably lower than the 61.5% reported by Gallup and Norris (1939 c). They also noted that Gallup and Norris (1939b) fed a hen diet with 200 ppm Mn. Yair and Uni (2011) confirmed that the yolk is a primary storage depot for Zn, Mn, and Cu but they also observed considerable Zn (37%) and Cu (23%) storage in the albumen vs. only 3% for Mn. These authors reported a progressive decline in yolk content (*ug* or mg/yolk) between ED 0 – ED 11, a somewhat accelerated decline from ED 11- ED 17 with very little additional disappearance from ED 17 through hatch. Total consumption (disappearance) between set and hatch was 94.2% (Zn), 86.7% (Mn) and 95.5% (Cu). More recently, Hopcroft et al. (2019) reported similar patterns of yolk disappearance or uptake from ED0 to ED 17.5 although their reported baseline level of Mn (*ug*/yolk) was reached at ED 13.5. These authors also reported considerable differences in the yolk content of Zn, Mn, and Cu vs. what was observed by Yair and Uni (2011) and they emphasized the importance of the maternal diet when comparing different reports on TM dynamics during the course of incubation. With respect to hen age and source of dietary TM, Favero et al. (2013) observed significant hen age effects on yolk/albumen Zn, Mn, and Cu concentrations similar to what was reported by Kienholz et al. (1964) for Zn. The TM in hen diets were supplemented as inorganic sulfates or amino acid chelates and there were no diet (source) effects on Mn or Cu concentration nor any diet by age interactions for any of the minerals. There was a significant hen age effect on tibia length, dry weight and percentage ash at hatch but no TM source effects or age by source interaction.

### Embryonic In Ovo Modulation

In the poultry industry, innovation often takes the form of a unique process that is often facilitated by established industry practices. The development of in ovo injection technology is one example of this because the concept and ultimately commercial application were compatible with the manual transfer of eggs to a “hatcher” at approximately 18 days and post-hatch vaccination for Mareks disease in the hatchery. The manual vaccination alone was time consuming, labor intensive, and always had the potential for additional chick stress prior to placement (Gildersleeve et al., 1993). The avian immunology research and agricultural engineering underlying the feasibility of in ovo vaccination resulted in numerous patents prior to its successful introduction to the industry (Miller, 1984; Sharma and Burmester, 1984; Hebrank, 1987). In addition to its continued use for Mareks vaccination, research subsequently led to the development of additional in ovo viral vaccines and one for coccidiosis (Thaxton, 1994; Reynolds, 1998; Evans et al., 2002).

The near universal acceptance and benefits associated with in ovo vaccine delivery has generated considerable research interest in the potential for in ovo delivery of nutraceuticals and other physiological modulators (Tako et al., 2004; Uni and Ferket, 2004; Uni et al., 2012). The aforementioned decline in yolk TM through ED17 of incubation was the impetus for studies on late term in ovo mineral supplementation. Yair and Uni (2011), using in ovo supplementation at ED 17, were successful at eliciting small but significant increases in yolk sac Fe, Zn, and Cu levels (*ug*) at ED20 and Hatch (ED21) and considerably greater increases in Mn at the latter 2 time points. Yair et al. (2013), using the same in ovo treatments, observed a consistent increase in tibia Mn from ED 19 through 7 d post-hatch with no effects on Zn. There were no consistent treatment effects on tibia or femur length or weight from ED 19 through 54 d post-hatch, structural properties of cortical bone (cortical area, medullary area, crosssectional thickness) or indices of mineralization (cortical bone ash, bone mineral density). It should be noted that cortical bone samples from the midshaft region have ash values that are considerably higher than if the “whole bone” was sampled. These authors conducted a subsequent study which included both an inorganic and an organic TM treatment with vitamin D_3_ in the mix of enrichment nutrients (Yair et al., 2015). There were no differences between TM sources and both Enrichment treatments significantly increased Cu, Zn, and Mn yolk content to levels much higher than those reported earlier by Yair and Uni (2011). There were again, no Enrichment effects on BW, tibia weight or length and no differences in the structural properties of cortical bone before or post-hatch. At 38 d but not 10 d post-hatch, organic TM/ vitamin D_3_ Enrichment increased cortical bone ash 1.2% and 1.4% over 2 non-enriched control treatments, respectively, with no effects on bone mineral density. Cortical bone structure is a critical area with respect to “leg problems” associated with rapid growth in modern broilers (Williams et al. 2000, 2004; Rawlinson et al., 2009) and in ovo TM supplementation alone did not appear to modulate this. Oliveira et al. (2015) administered 2 levels of TM in ovo at ED17 and there were also no significant treatment effects on selected tibia traits at hatch. The percentage bone ash was significantly increased in the high TM treatment but neither treatment changed the proportion of TM in the bone ash from Enriched chicks.

In writing this review, it became apparent that in the early days of poultry nutrition research on TM and skeletal development, the Norris lab at Cornell was a consistent source of published research and future scientists. Over the last 25 years, it is safe to say the same could be said for the Uni lab and Hebrew University with respect to advancing our understanding of embryonic and post-natal intestinal development and the potential for in ovo nutrition.

### Manganese, Cartilage Matrix, and Endochondral Bone Growth

When Gallup and Norris (1938) conducted experiments with Mn adequate and deficient diets, samples were collected from chicks of the same sex, age, similar body weights and with no visible signs of perosis. The deficient chicks had noticeably shorter and thicker bones at 24 d with no visual differences in calcification. There was a short sentence near the end of the paper that would later prove to be very insightful: “*Partial depletion of the bones in manganese resulted in deformities at the joints and at the ends of the bones.”*These observations were subsequently confirmed by Shils and McCollum (1943) and Amdur et al. (1945) in rats and mice, respectively. In the latter report, rats fed the deficient diet had similar body weights, small but significant reductions in tibia length, bone density and a reduction in femur strength. Ellis et al. (1947) pair-fed rabbits either normal or Mn deficient diets to equalize intake and the deficiency decreased body weight as well as the length, density and percentage ash in the humerus.

Kay (1930) reported that in humans, plasma phosphatase was associated with bone disorders, particularly rickets while Wiese et al. (1939) would later hypothesize that phosphatase activity might be involved in the etiology of perosis. They fed chicks a perosis inducing diet developed by Clifcorn et al. (1938) and observed that blood and bone phosphatase activities were elevated at hatch and then declined to a steady state from 4 to 7 d post-hatch. The decrease in phosphatase occurred more quickly with Mn deficiency and remained elevated in chicks fed a supplemented diet (50 ppm Mn). A diet with added calcium phosphate (3%) but not calcium lactate reduced both blood and bone enzyme levels and these responses were negated with supplemental Mn. Using preps of bone phosphatase for in vitro studies, Ca and P ions both depressed enzyme activity although the negative effect of P was far greater. These effects were reversed with supplemental dietary Mn. In a subsequent report, Wiese et al. (1940) observed that the phosphatase concentration was proportionately greater than enzyme activity in normal bones (+ Mn) suggesting a role for Mn in both enzyme synthesis as well as activation. Combs et al. (1942) would later report that reduced phosphatase activity in chicks associated with Mn deficiency was correlated with reduced ash content in the tibia. Their observation that enzyme activity was much higher in the epiphyseal region vs. midshaft of long bones supported the visual observations of Gallup and Norris (1938). The reduction in phosphatase activity observed in Mn deficient chicks was not observed in deficient rats (Wachtel et al., 1943). Mohamed and Greenberg (1943) administered labeled Mn^56^ to both normal and Mn deficient chicks with perosis. Only the deficient chicks injected with labelled Mn had any accumulation in the bones and oral administration of the tracer was not detected in the bones of either control or deficient chicks. These authors concluded that the Mn requirement for normal bone development had to be quite small. In a subsequent study by Parker et al. (1955), radiolabeled Mn, Ca, and P was administered to Mn deficient chicks and there were no differences in the quantity or location of labelled Ca or P in the tibia. Deficient chicks, however, accumulated more labelled Mn with the highest concentration being detected in the epiphyseal region, near the site of active mineralization. These authors also reported that while bone mineralization was similar in both deficient and normal bones, the deficient bones were structurally weak and more prone to shattering when cut. Frost et al. (1959) would subsequently report abnormal epiphyseal cartilage formation in Mn deficient rats. For more detailed discussions on chondrocytes, cartilage and endochondral bone formation in chicks, we would direct readers to the following references (Leach and Gay, 1987; Roach and Shearer, 1989; Roach, 1997).

In embryos with the inherited “micromelia” skeletal anomaly, the shortened long bones had an increase in percentage bone ash and decreased bone matrix which led Asmundson (1942) to hypothesize that matrix synthesis and mineralization were independent traits. Wolbach and Hegsted (1952) noted that the pattern of epiphyseal cartilage replacement via vascular penetration in chicks is different than what occurs in mammals. When these authors fed Mn or choline deficient diets, there were noticeable changes including reduced cellular proliferation and the presence of abnormal matrix. The reported changes in the matrix had no effect on bone mineralization and they suggested that either Mn or choline deficiency resulted in similar lesions associated with the onset of perosis (Wolbach and Hegsted, 1953). The incidence of slipped tendons in commercial practice continued, however, after it was accepted that Mn was a causative factor in nutritional perosis. Thomas and Lowther (1976) compared commercial “field” broilers with slipped tendons with age matched experimental birds fed Control or Mn deficient diets. There were no differences in proximal tibia histology in the “slipped tendon” commercial or Control birds but Mn deficiency resulted in fewer proliferative cells and a disorganized columnar structure. There were no differences in the Mn content of epiphyseal cartilage sampled from Control and “slipped tendon” field broilers which was contrary to the suggestion by Wise et al. (1973) that all conditions resulting in “slipped tendons” had a common underlying mechanism.

The PhD dissertation of Leach (1960), a student of Norris, focused on TMs, specifically manganese, and the development of the epiphyseal growth plate in chicks. The dissertation research supported the hypothesis that Mn deficiency specifically affected the matrix mucopolysaccharide (proteoglycan) fraction in epiphyseal cartilage. Leach and Muenster (1962) would subsequently show that Mn but not choline deficiency significantly affected the proteoglycan composition of the matrix. Mn deficiency decreased the total hexosamine content of epiphyseal cartilage, largely due to a reduction in the galactosamine fraction. Both the total hexosamine and galactosamine matrix levels responded in a dose response manner to incremental Mn supplementation to a deficient diet. Leach (1968) would later observe that the reduction in size of the epiphyseal growth plate was the result of a reduction in matrix formation rather than decreased cell proliferation. While choline deficiency was effective at inducing a perosis like condition, there were no differences in the concentration of total hexosamines, specifically galactosamine, in cartilage taken from control or choline deficient chicks. Stock and Latshaw (1981) took a somewhat different research approach with incremental supplementation of manganese, choline or biotin to a basal control diet. Supplementation of each trace nutrient increased total hexosamines above what was observed for the basal diet in both articular and epiphyseal cartilage though the determination of individual proteoglycans was not reported.

Chondroitin sulfate is the primary proteoglycan in epiphyseal cartilage. It contains 2 galactose residues along with a xylose to form a trisaccharide that links linear polysaccharide chains with a serine residue on a core matrix protein. Leach et al. (1969) showed that 2 enzymes involved in the galactose transfer to the initial trisaccharide (galactosyl-transferase) and subsequent chain elongation with repeating disaccharide units (polysaccharide polymerase) require Mn as an essential co-factor. Liu et al. (1994) later showed that proteoglycan monomers from normal cartilage were almost exclusively chondroitin sulfate (92%) and keratan sulfate (8%). In Mn deficient cartilage, however, this fraction was significantly reduced (75%) with the appearance of a second fraction (25%) with a reduced carbohydrate content. The authors suggested this could represent either smaller or fewer side chains associated with the core protein. Leach and Gross (1983) also studied the effect of dietary Mn on the organic matrix of eggshells. There were morphological changes in the mammillary cores associated with Mn deficiency concomitant with a reduction in the hexosamine and hexuronic acid concentrations. levels. They also observed that the polysaccharide chains in normal eggshells were different than those extracted from cartilage proteoglycans.

### The Inorganic – Organic Trace Mineral Debate

Actual TM deficiencies have largely disappeared in commercial practice and more recent literature often cites one of the following reasons for continued TM research:

1. The almost 30 years since the last NRC requirement report was published (NRC, 1994).
2. The significant genetic gains in traits of economic importance and the concomitant pressure on skeletal development (Williams et al., 2000, 2004; Yair et al., 2017). Rarely is it mentioned, however, that indirect selection for feed intake has been a primary driver in the genetic gains in BW (Chambers et al., 1981) and this would increase TM intake in the absence of increased dietary content.
3. The emergence of commercially available organic TM and the differences in bioavailability compared with traditional inorganic salts (Richards et al., 2010).
4. The use of modern molecular tools that have identified potentially new mechanistic roles for selected TM (Liu et al., 2015; Lu et al., 2016).

Suffice it to say, both the organic and inorganic segments of the TM market each include multiple products with their own individual pros and cons. With respect to inorganic TM, one of the first comparison studies was Gallup and Norris (1939a) who reported that all 5 inorganic Mn sources tested (MnCl_2_, MnSO_4_, KMnO_4_, MnCO_3_, MnO_2_) were equally effective in decreasing the incidence of perosis in diets with 50 ppm Mn. Hennig et al. (1966), as cited by Watson et al. (1971), subsequently showed that whole body uptake of ^54^Mn from ^54^MnCl_2_ was greater than either ^54^MnSO_4_ or ^54^MnO_2_ in chicks dosed with the various isotopes. Watson et al. (1970, 1971) determined the solubility of different MnO sources compared with reagent grade MnSO4 (100%). The MnO solubilities in 0.4% HCl were 30 to 50% (Watson et al., 1970; n = 2 sources) and 2.7 to 87.2% (Watson, et al. 1971; n = 5 sources). Any differences in BW or bone ash between MnSO_4_ and individual MnO sources, however, were not a function of solubility differences. In both reports, MnSO_4_ was used in a dose response assay and the regression of bone ash or total bone Mn on intake showed the latter to be the best assessment of bioavailability. Subsequent studies by Black et al. (1984) and Henry et al. (1989) confirmed that bone Mn could be an effective tool for bioavailability studies. Reagent grade MnSO_4_ and MnO were used in both of the aforementioned studies with maximum levels set at 2,100 ppm and 4,000 ppm Mn above a basal diet (~ 100 ppm Mn). There were considerable differences in bone Mn bioavailability estimates for MnO (91%, Henry et al., 1989; 65.6%, Black et al., 1984) which emphasizes the variability that exists even among well designed studies. Edwards and Baker (1999) reported bioavailability estimates for analytical grade ZnSO_4_ and ZnO were similar and only slightly higher than 2 feed grade sources. An additional 2 feed grade ZnO sources were also tested, however, and their bioavailability estimates were less than 50% which supported an earlier report by Wedekind and Baker (1990b).

It is beyond the scope of this review to discuss the chemistry and bioavailability differences of organic and inorganic TM sources. Readers are directed to Cao et al. (2000) who did an excellent job discussing this topic for multiple organic Zn sources and zinc sulfate. As noted by Cao et al. (2000), not all organic TM have the same characteristics when exposed to varying pH environments within the intestine and these individual responses often become a talking point for a company's marketing efforts. These authors did report that bioavailability estimates of multiple organic Zn TM when compared to ZnSO_4_ ranged from little difference to a 30% improvement.

One last point that needs to be addressed is the potential environmental outcome of excessive TM supplementation to commercial diets. In a study by Bao et al. (2007), a basal sorghum-isolated soy protein diet was formulated with the following TM levels (4 ppm Cu, 15 ppm Mn, 20 ppm Zn) to which were added different quantities of an organic TM source or inorganic sulfates. A ‘mid” organic treatment level of TM supplementation (4 ppm Cu; 40 ppm Mn; 40 ppm Zn) optimized BW gain and feed conversion. This level of supplementation also significantly decreased TM excreta levels compared with 2 higher TM treatments which were closer to industry standards. The basal diet plus “mid” organic TM supplementation level was very similar to those levels recommended in the most recent NRC publication (NRC, 1994) which, as mentioned earlier, is often touted as being outdated.

## SUMMARY AND CONCLUSION

The intent of this review was to emphasize the significant contributions of research published in the early to mid-1900’s to our fundamental understanding of mineral/TM nutrition, nutrient interactions as related to intestinal digestion/absorption and skeletal development. Much of this research was published in some of the earliest issues of Poultry Science and during the 1930’s, the annual PSA meeting became a critical venue for the sharing of new data. This was an era in which biochemistry and nutrition research was often synonymous and Agricultural Biochemistry departments were common. This review has focused on Mn due to its importance in skeletal development.

An academic argument could be made that actual TM “requirements” have not changed substantially since they were originally established. Frank TM deficiencies are rarely seen but are routinely cited as rationales for the incorporation of organic vs. inorganic TM due to perceived improvements in digestive efficiency. For broilers and turkeys, significant genetic gains in commercially important traits when combined with changes in management paradigms (i.e., antibiotic free production) have certainly not eliminated the potential for skeletal weakness with bone infections (i.e., osteochondrosis) appearing to account for an increasing percentage of total skeletal anomalies. The last quarter century has also seen commercial development and widespread use of exogenous enzymes including phytases, carbohydrases, and proteases but their potential effect on dietary TM needs has received only token research attention.

## DISCLOSURES

This is an original review article and there is no conflict of interest.
